# Highly Sensitive and Wide-Dynamic-Range Multichannel Optical-Fiber pH Sensor Based on PWM Technique

**DOI:** 10.3390/s16111885

**Published:** 2016-11-09

**Authors:** Md. Rajibur Rahaman Khan, Shin-Won Kang

**Affiliations:** School of Electronics Engineering, Kyungpook National University, 80 Daehakro, Bukgu, Daegu 41566, Korea; rajibur@ee.knu.ac.kr

**Keywords:** pH, pH sensor, side-polished optical-fiber, sensing element, optical-fiber pulse width modulation, sensing membrane

## Abstract

In this study, we propose a highly sensitive multichannel pH sensor that is based on an optical-fiber pulse width modulation (PWM) technique. According to the optical-fiber PWM method, the received sensing signal’s pulse width changes when the optical-fiber pH sensing-element of the array comes into contact with pH buffer solutions. The proposed optical-fiber PWM pH-sensing system offers a linear sensing response over a wide range of pH values from 2 to 12, with a high pH-sensing ability. The sensitivity of the proposed pH sensor is 0.46 µs/pH, and the correlation coefficient R^2^ is approximately 0.997. Additional advantages of the proposed optical-fiber PWM pH sensor include a short/fast response-time of about 8 s, good reproducibility properties with a relative standard deviation (RSD) of about 0.019, easy fabrication, low cost, small size, reusability of the optical-fiber sensing-element, and the capability of remote sensing. Finally, the performance of the proposed PWM pH sensor was compared with that of potentiometric, optical-fiber modal interferometer, and optical-fiber Fabry–Perot interferometer pH sensors with respect to dynamic range width, linearity as well as response and recovery times. We observed that the proposed sensing systems have better sensing abilities than the above-mentioned pH sensors.

## 1. Introduction

Over the last several decades, the development and applications of chemical and biosensors have grown very rapidly [1]. Among chemical sensors, pH sensors have received the most attention because of the importance of pH measurement in biomedical, scientific, and chemical research; the food and beverage industries; diagnostic centers; the agricultural sector; etc.

In the biomedical research field, a pH sensor is used to monitor the pH in a microcell culture [2]. pH sensors are used in the food industry for quality control of food processing; in the fermentation process [3]; to determine the freshness of meat [4], milk quality [5], and the quality of drinking water [6]; and to measure microbial growth. In the medical field and at diagnostic centers, pH sensors are widely used to determine the acidity of body fluids [7,8] as well as blood pH [9]. In industrial sectors, pH sensors are used to process control in bioreactors [10] and to determine the presence of heavy metal ions in industrial wastewater. pH sensors are also used in environmental monitoring to determine the acidity of rain water [11].

Research studies have attempted to detect pH in the following areas: potentiometric [12], micro-electro-mechanical systems (MEMS) [13], capacitive [14], surface acoustic waves [15], complementary metal–oxide–semiconductor (CMOS) [16], carbon nanotubes [17,18], chemiresistors [19,20], red green blue (RGB) colors array [21], fluorescent [22], surface plasmon resonance (SPR) [23,24], localized surface plasmon resonance (LSPR) [25,26], etc. 

Optical-fiber sensors have become popular because of their acceptable cost, light weight, high accuracy, small dimensions, geometrical versatility, capability of monitoring remote sensing, nonexplosivity, immunity to electromagnetic interference, freedom from strong magnetic fields, and completely electrical isolation. Therefore, optical-fiber sensors are very safe, can detect multiple parameters using a single optical fiber without crosstalk, and can measure at inaccessible sites in harsh environments as well as from long distances. Many optical-fiber pH sensors have been developed based on different optical-fiber architectures, including the following: without/removed cladding [27], optical-fiber U-shaped pH sensor [28,29,30], side-polished optical-fiber pH sensor [31], D-type optical-fiber pH sensor [32], optical-fiber Bragg-grating pH sensor [33,34], and optical-fiber Fabry–Perot pH sensor [35]. 

An optical-fiber sensor that detects the pH of a fluid can be based on the principles of intensity modulation or wavelength modulation. For optical-fiber intensity modulation pH sensors, different types of pH-sensitive materials/dyes are used on the optical-fiber core or polished cladding. They measure the pH value of a given fluid by observing optical parameters such as absorption, reflection, transmission of optical power, and fluorescence. On the other hand, wavelength-modulated pH sensors use a pH-sensitive hydrogel with an optical-fiber Bragg grating [36], a long period grating [37], or a reflective diffraction grating [38]. Constructing intensity-modulation pH sensors is easy, but their sensitivity is low, and fluctuations in the light intensity affect the pH measurement. Wavelength-modulated pH sensors also have some disadvantages including a complex fabrication process, low sensitivity, and high complexity.

A potentiometric pH sensor utilizing different pH-sensitive polymers coated on wire electrodes was proposed by Santiago et al. [39]. The main feature of this sensor is its highly sensitive (approximately −43 mV/pH) and simple construction and linear sensing response. However, the proposed pH sensor also has disadvantages such as its bulk, a low pH measurement range of 3 to 10, and a high response time of approximately 3 to 5 min on average. 

Mishar et al. proposed an optical-fiber SPR sensor to detect the pH of fluid [23]. In this study, the researchers deposited different metal layers on an unclad optical-fiber using the thermal evaporation method. Then, they deposited a hydrogel layer on the deposited metal layer to fabricate an optical-fiber SPR sensor. Although the sensor’s construction is very simple and it can detect pH levels from 3 to 11, it has several disadvantages including high cost owing to different metal depositions, bulk, nonlinear response properties, and difficulty in determining the resonance wavelength.

A pH sensor using a shear horizontal surface acoustic wave was proposed by Haekwan et al. in 2013 [15]. In their study, they prepared input and output interdigitated transducers on a piezoelectric substrate and placed a ZnO film containing nanoparticles as a pH-sensitive material between the input and output transducers. The construction and working principle of this sensor is simple, and it also gives a linear sensing response. However, its main disadvantage is that the pH measurement range is very low (from 2 to 7). 

Lei et al. proposed a graphene chemiresistor sensor to measure the pH of fluid [20]. The sensor has some advantages including ease of fabrication, small size, and good sensing ability. However, the proposed sensor has a low dynamic range of 4–10, and its performance is less stable. 

Gu et al. proposed and developed an optical-fiber sensor to measure pH in a range of 2 to 11 using a modal interferometer method [40]. They applied a layer-by-layer electrostatic self-assembly technique to deposit two kinds of polyelectrolytes on the side surface of a thin-core fiber modal interferometer. The sensors have several features including a wide pH sensing range, linear sensing response, and good stability. However, this sensor also has several disadvantages including a complex fabrication process, low sensitivity, and long response and recovery times (about 60 s and 80 s, respectively). 

An optical-fiber Fabry–Perot interferometer sensor for measuring pH was presented by Zheng and coworkers in 2016 [35]. The interferometer was prepared by placing a small section of a hollow-core photonic crystal fiber between a sensing and lead-in single-mode optical-fiber. The other end of the sensing fiber was deposited in a polyvinyl alcohol/poly-acrylic acid hydrogel containing a sensing membrane. The sensitivity of the proposed sensor was good, about 11 nm/pH, and offered good repeatable performance and sensing stability. The sensor had several disadvantages including a complex fabrication process, a low dynamic range of pH from approximately 4.1 to 6.9, nonlinear response properties, and high response and recovery times of approximately 60 s and 90 s, respectively. 

In our study, we proposed a fast, highly sensitive, wide-dynamic-range side-polished optical-fiber pH sensor array that is based on optical-fiber pulse width modulation (PWM) [41,42] principles. According to the optical-fiber PWM technique, the pulse width of the received sensing signal from the optical-fiber pH-sensing element of the array changes if the sensing element of the array comes into contact with a pH buffer solution. This can occur because of changes in the refractive index of the sensing membrane of the optical-fiber sensing element. In our experiment, five different kinds of pH sensitive dyes (methyl red, methyl orange, thymol blue, Nile red, and rhodamine-B) were used as the principal materials of the sensing membrane. These dyes were individually mixed with N,N-dimethylacetamide (DMAC) and Polyvinyl chloride (PVC) and deposited on side-polished optical-fiber devices to fabricate five optical-fiber sensing elements of an array. To observe the sensing ability of the proposed optical-fiber pH sensor array, we used buffer solutions of different pH (from 2 to 12), and we obtained a linear sensing response with highly stable response properties. The proposed optical-fiber PWM pH sensor has several other features: simple construction, ease of fabrication, low cost, reusability, and light weight. The sensor used electronic circuitry prepared from inexpensive available electronic components at local electronic components shops. We compared different sensing parameters of the proposed optical-fiber PWM sensor array with different pH sensors, and found that the proposed sensing system has a better sensing ability. 

## 2. Theory and Working Principle of the Optical-Fiber PWM pH-Sensing System 

**Figure 1:**
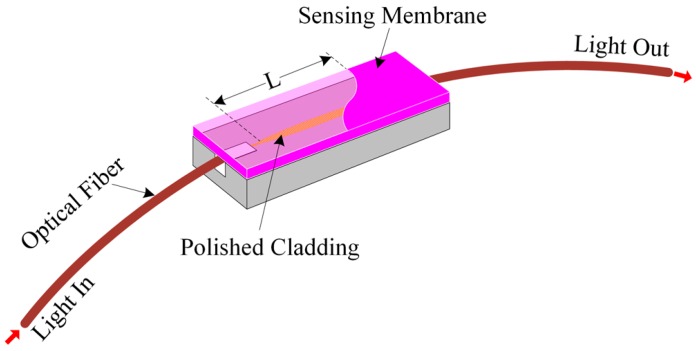
Schematic diagram of the side-polished optical-fiber with a sensing membrane.

We proposed an optical-fiber PWM-based pH-sensing system. Generally, an electrical PWM system consists of two inputs and an output. One input is called the pulse input, which is used for the electrical pulse entering into the system. The other input is called the control input, which is used to change the pulse width of the input signal without changing the time period of the input signal. The output is used to obtain a desired pulse width with the same time period of the input signal. In our experiment, we deposited a polymer waveguide that contained pH-sensitive dye on a side-polished optical-fiber device to prepare an optical-fiber pH-sensing element, as shown in Figure 1. 

**Figure 2:**
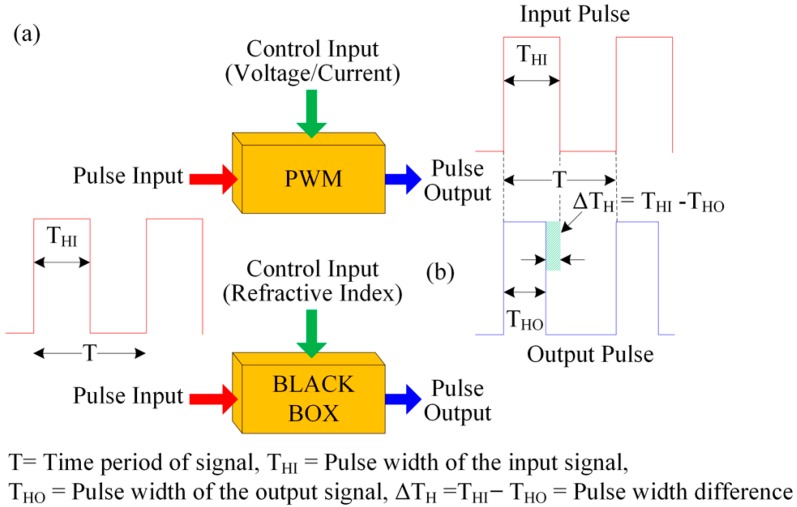
Functional analogy between the two PWM systems: (**a**) electrical; and (**b**) optical-fiber PWM sensing systems.

In the proposed optical-fiber PWM system, a light pulse is passed through a fiber-optic-based waveguide. When the sensing membrane of the optical-fiber sensing element makes contact with the pH buffer solution, then the optical properties (such as the refractive index of the sensing membrane) change. This in turn changes the pulse’s peak value as well as the fall time. This occurs as a result of the absorption of light in the waveguide. As a result, the pulse width of the received sensing signal changes. The pulse width of the received light pulse depends on light absorption into the polymer waveguide, which can be considered a pulse control input. The width of the light pulse is a result of the variation in the refractive index of the overlay waveguide, which corresponds to the change in pH of the buffer solution. Therefore, some analogies can be established between the electrical as well as optical-fiber PWM sensing systems with regard to their operating principles and structures. These analogies are presented in Table 1 and Figure 2. 

The purpose of Table 1 is to understand easily the analogies between the electrical and the proposed optical-fiber PWM sensing system. For example, the proposed optical-fiber PWM sensing system has three ports (in, control and out), which is similar to electrical PWM system. Moreover, in an electrical PWM system, a voltage/current is applied to its control port to change the pulse width of the output electrical signal, which is obtained at the out port. This is similarly found in the proposed optical-fiber PWM system. In the proposed PWM system, the control port is the change of refractive index of the sensing membrane of the sensing element. Therefore, if the refractive index of the sensing membrane change due to the change of pH value, then the out port light pulse’s width change without changing the time period of the in port light pulse. 

In our study, we polished down to the core on one side of the optical-fiber. Then a sensing membrane was deposited on this side-polished optical-fiber to create two waveguides. Therefore, when light passed through the side-polished optical-fiber, a fraction of the radiation extended a small distance (called an evanescent field) from the polished region. This evanescent field entered the upper waveguide from the side-polished optical-fiber waveguide. The evanescent field’s energy may change owing to absorption or scattering of light into the overlay waveguide, or from changes in the refractive index of the overlay waveguide. Mathematically, the evanescent field can be represented by [43]

(1) $$E\left(z\right)=E_{0}\exp\left(-\frac{z}{d_{P}}\right),$$

where $E_{0}$ is the electric field amplitude of light at the interface of core–cladding and z is the distance of electric field in the cladding from the interface. $d_{p}$ is the penetration depth, and the sensitivity of the side-polished optical-fiber sensor depends on the penetration depth. The penetration depth can be defined mathematically as [44]

(2) $$d_{p}=\frac{\lambda }{{2\pi n}_{1}}{\left\{\sin^{2}\theta -{\left(\frac{n_{2}}{n_{1}}\right)}^{2}\right\}}^{-0.5},$$

where λ is the wavelength of the transmitted light, and θ is the angle of incidence to the normal at the interface. $n_{1}$ and $n_{2}$ are the refractive index of the fiber cladding and the material of the overlay waveguide, respectively. Now, if a light pulse with time period T is transmitted through the side-polished optical-fiber waveguide, then the received light pulse width $T_{H}$ can be written mathematically as [41,42]

(3) $$T_{H}=\frac{T}{n_{2}{\left(\frac{\gamma L}{\alpha }\right)}^{0.5}},$$

where L is the length of the polished cladding, γ is the evanescent wave absorption coefficient, and α is the phenomenological ion-specific parameter. Therefore, by observing the pulse width of the received sensing signal, the pH-value can be determined. The pulse width is proportional to the pH, which corresponds to changes in the refractive index of the overlay waveguide as well as the absorption of the evanescent field into the waveguide.

## 3. Experimental Details

### 3.1. Fabrication of the Side-Polished Optical-Fiber Device

**Figure 3:**
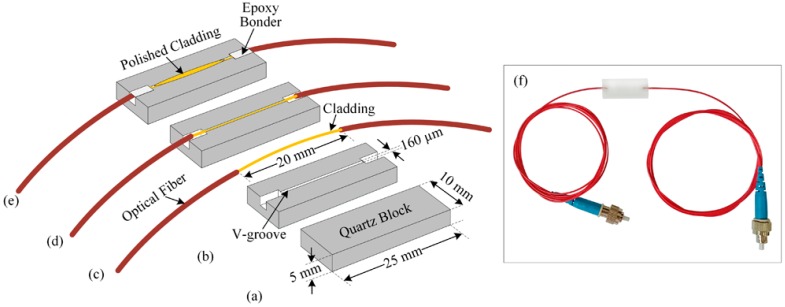
Fabrication process for side-polished optical-fiber device: (**a**) quartz block; (**b**) making the V-groove in the quartz block; (**c**) removing the jacket of the optical-fiber; (**d**) bending the portion of the optical-fiber with jacket removed, placing it into the V-groove of the quartz block and apply epoxy; (**e**) polishing the optical-fiber quartz block; and (**f**) photograph of fabricated side-polished optical-fiber device.

In our study, we fabricated a side-polished optical-fiber device for preparing the optical-fiber pH sensing element of the array. To do this, we chose a quartz block of approximately 25 × 10 × 5 mm^3^, and made a V-groove of approximately 160 µm in width using a mechanical slicer. Then, we collected a single-mode optical-fiber of about 1 m in length and removed its jacket approximately 20 mm at the middle. The radius of the optical-fiber core was 3 µm, and the cladding radius was 125 µm. The removed jacket portion was bent with a radius approximately 60 cm and placed in the V-groove of the quartz block. Then, we applied and dried epoxy so the bent cladding portion was strongly attached to the quartz block. The surface of the cladding attached to the quartz block was polished using l000-μm and 8000-μm polishing powders on polishing pads to fabricate a side-polished optical-fiber device. Figure 3a–e shows the step-by-step fabrication procedure for the side-polished optical-fiber device, and Figure 3f shows a photograph of the prepared side-polished optical-fiber device. 

### 3.2. Fabrication of the Sensing Membrane and Optical-Fiber Sensing Element

To make optical-fiber pH sensing elements of the array, we needed some pH sensitive chemicals which will change their optical properties such as refractive index and chemical structures when those dyes/chemicals containing polymer waveguide of sensing elements in contact with the pH buffer solution. Those dyes/chemicals change their molecular and electronic structures in acidic, neutral, and alkali solutions, and the way of change their molecular and electronic structure is different. As a result, the dipole moment of the molecule changes, which in turn changes the dielectric constant as well as change the refractive index, since the overly sensing membrane contains pH sensitive dye. Therefore, when the sensing membrane of optical-fiber pH sensing element of the array contact with a pH buffer solution, the refractive index of the sensing membrane changes. As a result, the pulse width of the received sensing signal also changes. In our study, we used five different kinds of pH sensitive dyes (methyl red, methyl orange, thymol blue, Nile red, and rhodamine-B) [45,46] to prepare five optical-fiber pH sensing elements of an array. We used five different dyes containing sensing elements of the array to observe the sensing ability of different dyes containing optical-fiber pH sensing element. We have chosen these five dyes because they are low cost, easy to make sensing membrane, and available in the chemical markets which also save our time. All dyes have different pKa values. To prepare the optical-fiber pH sensing element of the array, different dyes with different amounts were individually mixed with DMAC and PVC to create five different types of pH sensing solution. The chemical compositions of the five different pH-sensing solutions for different optical-fiber pH-sensing elements (S1 to S5) of the array are tabulated in Table 2. The preparation procedure for the pH sensing solution was as follows: first, we mixed different amounts of dye individually with 4 mL of DMAC solution and sonicated it for approximately 10 min to make the dye solution. Then, we added 0.30 g of PVC to the dye solution to make a pH-sensing solution. All chemicals were collected from the Sigma-Aldrich Chemical Corporation (Seoul, Korea) and used without further purification. We cleaned the polished area of the optical-fiber device properly with methanol, ethanol, and deionized (DI) water. Then, we dried the side-polished optical-fiber device using N_2_ gas. Next, we deposited different sensing solutions individually on the optical-fiber device using a spin coater with speed 1000 rpm, and dried them by placing them on a hotplate at 50 °C for 20 min to make five optical-fiber pH-sensing elements of the array. In our experiment, we used the PVC polymer to immobilize the pH sensitive dye properly on the surface of side-polished optical-fiber device. The thickness of the sensing membrane of the optical-fiber pH sensing element of the array was about 22 µm and was measured by a scanning electron microscope (SEM) (S-4800, Hitachi, Ibaraki, Japan). The relative standard deviation (RSD) of thickness of the sensing membranes was about 0.001.

### 3.3. Detection Mechanism of the Proposed Optical-Fiber pH Sensing System 

**Figure 4:**
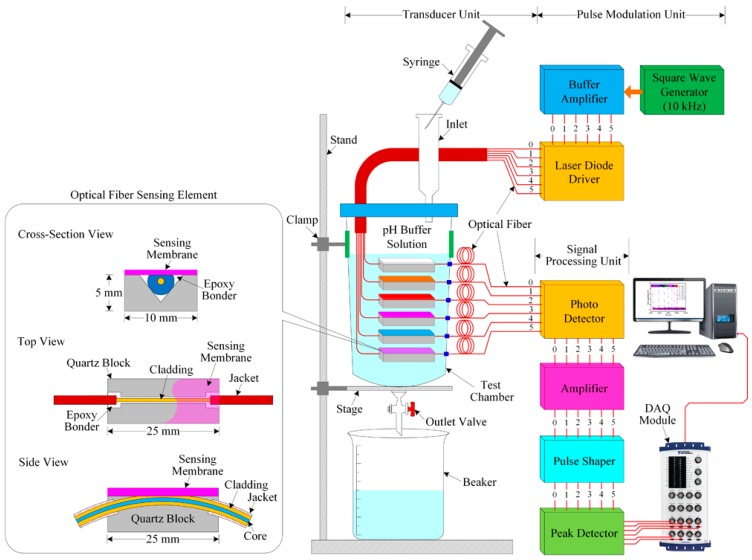
Schematic diagram of the experimental setup of the proposed optical-fiber PWM pH sensing system.

The experimental setup of the proposed optical-fiber PWM pH-sensing system is shown in Figure 4 and consists of three units: a pulse modulation unit, an optical-fiber pH sensor array used as a transducer unit, and a signal processing unit. We designed the pulse modulation unit and the signal processing unit using low-cost and easily available components from local electronics suppliers.

The pulse generator/modulation [47,48,49] unit consists of three units: a square-wave generator, a buffer amplifier, and a laser diode (LD) driver with LD. The square-wave generator produces a square wave with a frequency of 10 kHz and a 50% duty cycle. In our experiment, we designed a square-wave oscillator using a well-known timer (IC NE555) and associated electronic components. This square-wave generator offers a square wave with a frequency of 20 kHz without a 50% duty cycle. Our target is to obtain 10 kHz with a 50% duty cycle. Therefore, the output of the square-wave oscillator is fed to the input of a T-flip-flop, which consists of a CD4027 (JK flip-flop employed in toggle mode) to obtain a perfect 10-kHz signal with a 50% duty cycle. 

The output of the T-flip-flop is connected to the input of the buffer amplifier. The purpose of the buffer amplifier is to reduce loading effects because the buffer amplifier has low input impedance and high output impedance. Then, its output is fed to the input of the LD driver circuit, whose output is connected to an LD. The function of the LD driver circuit is to turn on/off the LD according to the pulse of its applied input signal. As a result, the LD emits a light pulse with a 10-kHz frequency at 850 nm. The LD is connected to the optical-fiber pH-sensing element. In our study, we used six LDs to transmit light pulses through the sensor array, which consists of five optical-fiber pH-sensing elements and one reference optical-fiber element. The opposite terminal of the pH sensor array is connected to six photodiodes. 

The signal processing unit consists of a photodetector (PD), amplifier, pulse-shaping circuit, and peak detector. The photodiode circuit converts the optical light pulse coming from the optical-fiber pH sensor array into an electrical pulse. The output of the PD circuit is connected to the input of the operational amplifier, which is connected to the current-follower configuration for desired signal amplification. The pulse-shaping circuit is used to shape the pulse as a square without distorting the signal, and its output is the input of the peak detector. The peak value of the signal is obtained from the peak detector. The six outputs of the peak detector are connected to the six inputs of the data acquisition (DAQ) module (NI USB-6216 BNC, National Instruments, Debrecen, Hungary). A computer is connected to the DAQ module. To observe the sensing performance of the optical-fiber pH sensor array and store the data in the computer, we developed a LabVIEW program.

An amplitude modulation based side-polished optical-fiber sensor cannot detect a very small change in the light due change of refractive index of the overlay sensing membrane. However, in the case of the proposed optical-fiber PWM sensing system, the received light pulse width depends on the light pulse amplitude as well as fall time. The effect on the pulse width amplitude and the fall time caused by a small change of refractive index of the overlay sensing membrane. As a result, the proposed optical-fiber PWM pH sensing system offers a good linear dynamic range and can effectively detect low pH. A laser light source with higher wavelength is required to obtain better sensitivity and we obtained better sensing performance using the 850 nm laser source, which is why in our experiment we have selected the laser diode of 850 nm wavelength.

The sensing response of a specific sensing element is the pulse width differences between the signals which is received from the reference element and that particular sensing element of the array. In our experiment, before measuring the pH of the buffer solution, we calibrated the system so that the same amount of light pulse passes through all optical fiber-sensing elements as well as a reference element. As a result, we obtain a pulse width of zero. The relative pulse width (ΔT_H_) of a given pH is the difference between the reference pulse width and the sensing pulse width. Then, we slowly inject pH buffer solution by a syringe into the test chamber, and observe the sensing response at room temperature. The fabricated optical-fiber sensor array carries different pH-sensitive compounds. Therefore, when the sensor array makes contact with any buffer solution, the refractive index of the sensing membrane changes. As a result, the width of the light pulse corresponds to changes in the received electrical signal pulses as well as changes in the output voltages of the peak detectors. The pulse width of the received signal depends on the amount of pH and the properties of the sensing membrane’s materials. The relative pulse width increases as the pH of the buffer solution increases. An oscilloscope (OWON, VDS3104, Zhangzhou, China) is used to measure the pulse width of the received sensing signals. The thickness of the dye containing PVC polymer sensing membrane of the optical fiber sensing element was very thin and we also used the modulated light of frequency about 10 kHz, which increase the fast sensing response to changes in pH. In our experiment, we obtained the higher sensitivity and faster sensing response at 22 µm thickness of the sensing membrane and the modulated light of frequency about 10 kHz. Rhodamine-B is a xanthene dye and its optical properties change under different pH values [50]. To observe the pH sensing ability of Nile red and rhodamine-B under different pH, we prepared Nile red and rhodamine-B containing pH solutions. To do this, Nile red and rhodamine-B were individually dissolved into different pH (2–12) of buffer solution to make 10 mM of Nile red and rhodamine-B containing pH solution, then we observed the absorption peak of those dyes containing pH solutions and we found that as the pH of a Nile red/rhodamine-B containing pH buffer solution increases, the absorption peak of the Nile red/rhodamine-B containing pH solution increases as well. The experimental setup of this experiment is discussed in detail in [48]. This occurs owing to a change in the refractive index of dye containing pH solution. These results indicate that the Nile red and rhodamine-B can produce responses for a range of pH. The absorption (a.u.) peak of rhodamine-B containing pH 2, pH 4, pH 8, pH 10, and pH 12 solutions was about 4676.17, 5442.27504, 5691.28, 6042.16, and 6475.09, respectively. Moreover, a colorimetric pH sensor array including Nile red was reported in [45]. The main feature of the proposed sensor is its high sensitivity, stability, fast response time, and its principle of operation is called the optical-fiber PWM technique, which was proposed by us and it was reported first time to develop pH sensing system using the PWM technique by this paper. The proposed optical-fiber PWM pH sensing system is suitable to detect pH of any sample solution in agriculture, environment, medical, research sectors and so on. 

## 4. Results and Discussion

**Figure 5:**
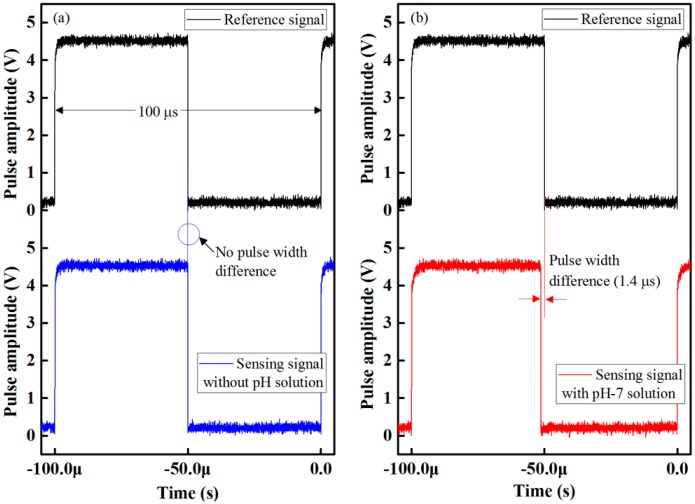
Waveform response of the proposed optical-fiber PWM pH-sensing system: (**a**) without buffer solution; and (**b**) with buffer solution at pH 7 for methyl red containing optical-fiber sensing element of the array.

The waveform response of the proposed optical-fiber PWM pH-sensing system is shown in Figure 5a. There were no pulse-width differences between the reference and sensing signals when there was no buffer solution in the test chamber. However, when we injected a buffer solution into the test chamber, the pulse width of the received sensing signal decreased. As a result, the relative pulse width between the sensing and the reference signals increased. This is shown in Figure 5b. The relative pulse-width difference of the buffer solution at pH 7 for methyl red, which contained an optical-fiber sensing element of the array, is about 1.4 µs. We also observed the response of the proposed PWM sensing system after removing the buffer solution of pH 7 and we found that as the buffer solution is removed from the test chamber the pulse width of the received sensing returns to its original state in less than 12 s and we observed that there is no pulse width difference between the sensing and the reference signal after removing the buffer solution from the test chamber. This result indicates that the sensing performance of the designed signal processing unit is excellent, and that it has the ability to detect small differences in the pulse width.

**Figure 6:**
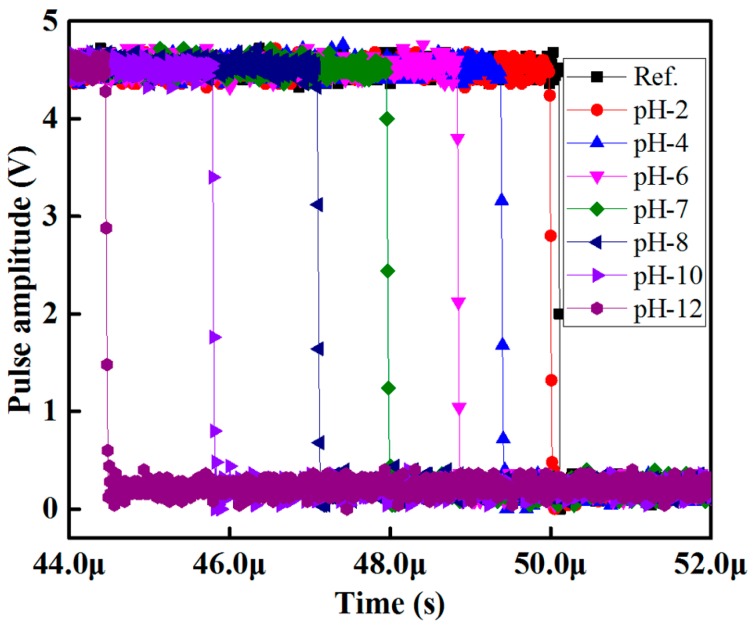
Pulse width response of the proposed optical-fiber PWM pH sensor array for methyl red containing sensing element S1.

The pulse-width differences between the sensing signal and the reference signal in a buffer solution of pH 2 to 12 for methyl red containing optical-fiber sensing element S1 of the array is shown in Figure 6. In Figure 6, it is seen that the relative pulse width increases as the pH-value of the buffer solution increases.

**Figure 7:**
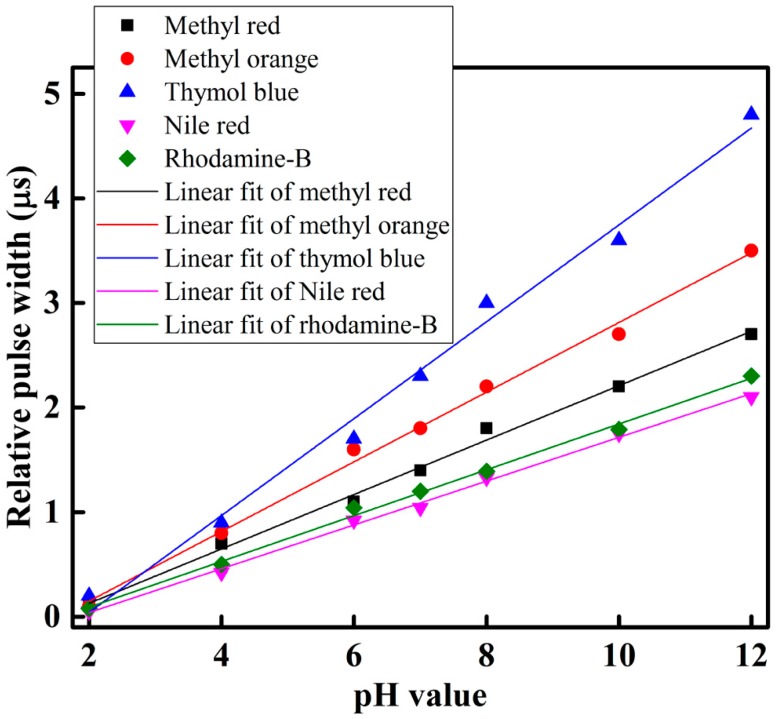
pH response of the proposed optical-fiber sensor array.

**Figure 8:**
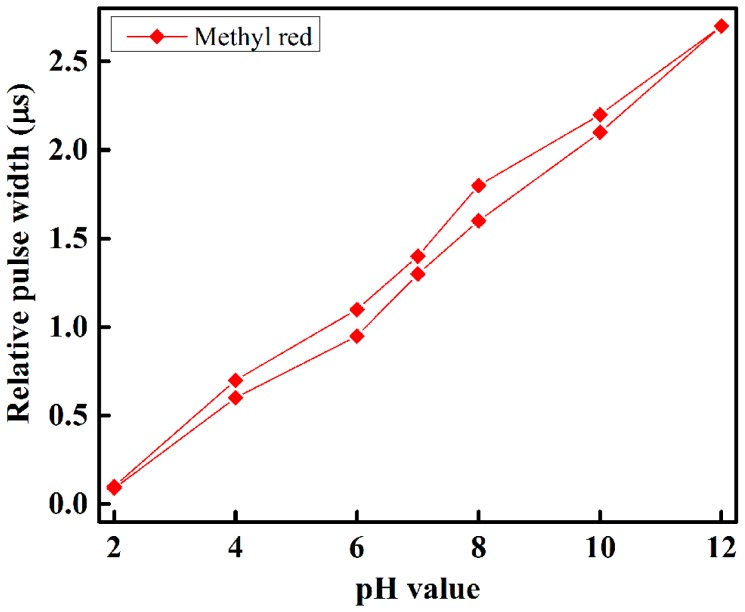
The hysteresis response of methyl red containing sensing element of the array.

To observe and determine the performance of each sensing element of the optical-fiber sensor array with respect to different pH-value of the buffer solution, we slowly injected the buffer solution with different pH-value individually in the test chamber, and took measurements at room temperature. The sensing performance of the proposed optical-fiber PWM pH-sensing system of five sensing elements of the array for a buffer solution of pH 2 to 12 is shown in Figure 7. This figure shows that as the amount of pH in the buffer solution increases, the relative pulse width increases linearly. A linear curve fitting is used in Figure 7 to determine the slope, i.e., the sensitivity of the proposed optical-fiber PWM pH-sensing systems. According to our experimental observations, it is found that the sensing element of the array offers a linear sensing performance over the wide dynamic range. It also indicates that the highest detection corresponds to thymol blue containing sensing element S3, while the lowest detection rate corresponds to Nile red containing sensing element S4 of the optical-fiber sensor array. The hysteresis response of the proposed optical-fiber PWM pH sensing system for methyl red containing sensing element of the array is shown in Figure 8.

The proposed highly sensitive pH sensing system is based on the optical-fiber PWM principle and the received sensing signal pulse width change due to a small change in refractive index of the sensing membrane. This refractive index of the sensing membrane changes due to changes in the optical properties as well as the chemical structure of dye. For example, thymol blue shows red, yellow, and blue color at pH 2, pH 7, and pH 12, respectively. From this response, we can see that thymol blue changes its refractive index at different pH of buffer solutions, which corresponds to changes in the optical properties as well as the chemical structure. Therefore, when the thymol blue (as well as other pH sensitive dyes) containing sensing membrane of the optical-fiber sensing element in contact with any pH buffer solution, the refractive index of the sensing membrane changes as a result of the light pulse amplitude as well as fall time changes, which correspond to changes in the pulse width of the received sensing signal and offer a linear and wide sensing range.

**Figure 9:**
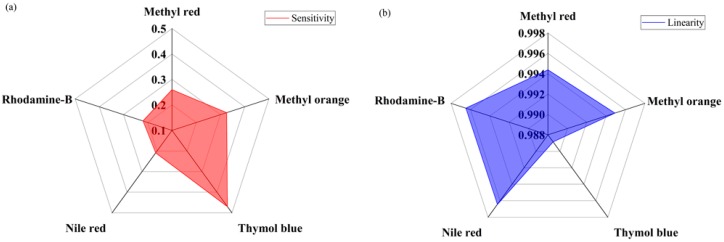
Performance of the proposed optical-fiber pH-sensing system: (**a**) sensitivity; and (**b**) linearity.

The radar chart in Figure 9a shows the sensitivities of the five optical-fiber sensing element of the proposed PWM pH-sensing systems under different pH of the buffer solution. According to the results, the proposed sensing systems offer the highest sensitivity for thymol blue containing sensing element S3, whose sensitivity is about 0.46 µs/pH. The lowest sensitivity is offered by the Nile red containing sensing element S4, with a sensitivity of about 0.21 µs/pH. 

Linearity is an important parameter of the sensor and it is represented by the correlation coefficient R^2^ value. If the R^2^ value is near 1 it indicates that the performance of sensing system is more linear. The linear performance of the proposed PWM pH-sensing system is presented in Figure 9b. According to the experiment, the fifth sensing element S5 of the array that contains rhodamine-B with a sensing membrane offers the highest linearity, with a correlation coefficient R^2^ of approximately 0.997. The lowest linearity is offered by the third sensing element S3 of the optical-fiber sensor array, and its R^2^ is approximately 0.988. In our experiment, the third sensing element, S3, which contains thymol blue, shows the highest sensitivity and lowest linearity of the sensing elements in the array. If we look at Figure 7, we can see that, in the case of thymol blue, some measuring points are shifted from the mean value, which increased the variance as a result the linearity, i.e., R^2^ value decreased. 

In our experiment, we determined the precision/reproducibility performance of the proposed optical-fiber PWM pH-sensing system. Therefore, we prepared five samples of methyl red containing an optical-fiber sensing element and observed the sensing ability of these optical-fiber sensing elements in a buffer solution with a pH of about 7. According to our experiment, we found that the five methyl-red-containing optical-fiber sensing elements had almost the same sensing performances. Table 3 lists the statistical data for those five measurements. Therefore, we can say that the sensing elements have excellent precision/reproducibility performance, and their relative standard deviations (RSDs) were about 0.019. 

In our study, we used five different dyes containing optical-fiber sensing element of the array and we observed the precision/reproducibility performance of all sensing elements of the array and the performance were almost same. Therefore, in this study, we only represent the reproducibility performance of the methyl red containing pH sensing element S1. The reproducibility performances of methyl orange, thymol blue, Nile red, and rhodamine-B containing pH sensing elements were 0.020, 0.0195, 0.021 and 0.0199, respectively.

The performance of the proposed optical-fiber PWM pH sensing system was tested for four months and used many times to observe the change of the properties of membrane as well as sensing element’s properties. We found from the collected data after this extended period that the sensing element offered almost the same sensing response. Therefore, we can say that the sensor has long-term stability and there are no sensing membrane/dye degradations of time. The standard deviation of stability of the proposed optical-fiber PWM pH sensing system was about 0.018 and the resolution of the proposed optical-fiber PWM pH sensing system was about 0.012 pH. The pulse width of the proposed optical-fiber PWM sensing system change due to change the refractive index of sensing membrane and organic fluorophores photobleach is not an issue for the sensing system.

**Figure 10:**
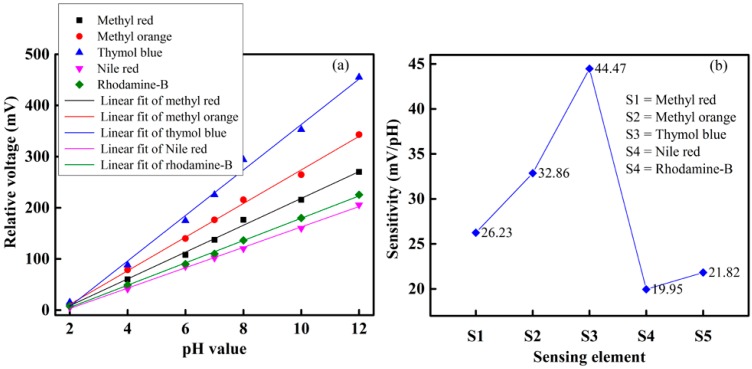
Sensing performance of the proposed optical-fiber pH-sensing system: (**a**) sensing ability; and (**b**) sensitivity.

The relative voltage response of the proposed optical-fiber PWM pH-sensing system in a buffer solution of pH 2 to 12 is shown in Figure 10a. It is found that the relative voltage of the proposed pH-sensing system increases as the pH of the buffer solutions increases. In Figure 7 and Figure 10, we have presented the relative pulse width and relative output voltage of the proposed pH sensing system under different pH of buffer solutions, respectively. Since the reference element of the array does not show any sensing response under a pH of buffer solution, it is used to compensate for common error sources such as environmental temperature and pressure. Therefore, in Figure 7 and Figure 10, we did not present the response of the reference element of the array.

**Figure 11:**
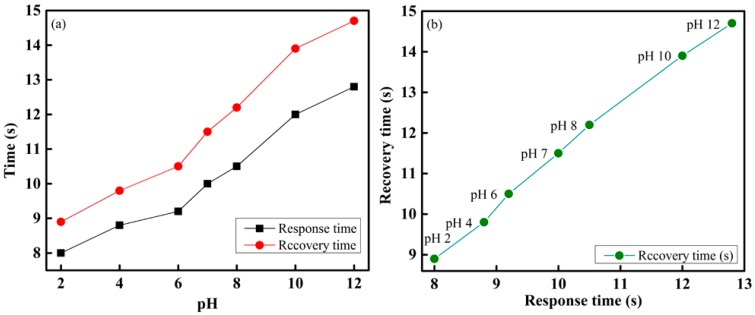
Performance of the fiber-optic PWM pH-sensing system: (**a**) response and recovery times; and (**b**) response versus recovery times of different pH of buffer solution.

Figure 11a shows the response and recovery times of the proposed optical-fiber PWM sensing system for sensing element S1, which contains a methyl-red sensing membrane. It is observed that the response and recovery times were approximately proportional to the increase in pH of the buffer solution. This occurs because at a high pH, the active sites on the surface of the sensing element are saturated. Therefore, in a buffer solution with a high pH, the reaction on the surface gradually gained control and slowed down the response and recovery processes. The response and recovery times of the proposed optical-fiber pH-sensing system were 8 s and 9 s, respectively. The response versus recovery times of the proposed pH-sensing system are presented in Figure 11b. 

In our study, we measured the unknown pH of buffer solutions using the proposed optical-fiber PWM sensing system and the commercially available pH-Ion meter (pH/Ion S220, Seven Compact, Mississauga, ON, Canada). First, we measured the pH of the unknown buffer solution using a pH-Ion meter S220. Then, we used our proposed system and compared the performance of the PWM system using the pH-Ion meter S220. To do this, we connected a voltmeter (Keithley, 2002, Cleveland, OH, USA) to the output terminal of the peak detector of the optical-fiber PWM sensing system. We calibrated the system using a known pH 7 in the buffer solution. The voltmeter read 7 mV, which corresponds to the pH 7 of the buffer solution. We also calibrated the pH-Ion meter S220 at a pH 7 of the buffer solution. Then, we took several measurements of unknown pH of buffer solutions. The results are tabulated in Table 4. From this measurement, we can say that the performance of the proposed optical-fiber PWM pH-sensing system was excellent. In our study, we tested the pH of body fluid (such as, urine) using the proposed optical-fiber PWM pH sensing system and we obtained an excellent response. We also have future plans to measure the pH of sea water. The proposed optical-fiber PWM pH sensing system is highly sensitive and two point calibrations are needed to obtain more accurate responses. 

We compared the performance of the proposed optical-fiber PWM pH-sensing systems with different pH sensors: potentiometric, optical-fiber modal interferometer, and optical-fiber Fabry–Perot interferometer with respect to several different sensing parameters. These parameters include pH detection range/dynamic range width, linearity, and response and recovery times. We observed that the proposed sensing systems have better sensing ability than the above mentioned pH sensors. 

Moreover, the dynamic ranges of the proposed optical-fiber PWM, potentiometric [39], optical-fiber modal interferometer [40], and optical-fiber Fabry–Perot interferometer [35] pH sensors were 2–12, 3–10, 2–11 and 4.1–6.9, respectively. Therefore, the dynamic ranges of the proposed pH-sensing system was wider than those of the other above-mentioned pH sensors. The response and recovery times of the proposed optical-fiber PWM sensor array were 8 s and 9 s, respectively. On the other hand, the response and recovery times of the potentiometric [39], optical-fiber modal interferometer [40], and optical-fiber Fabry–Perot interferometer [35] pH sensors were 3 and 5 min, 60 s and 80 s, and 60 s and 90 s, respectively. In addition, the linearity (i.e., the correlation coefficient R^2^) of the proposed optical-fiber PWM pH-sensing system was 0.997, which was higher than those of the above-mentioned pH sensors. 

## 5. Conclusions

In this paper, we presented a highly sensitive sensor array with a wide dynamic pH range. The array is based on an optical-fiber PWM technique. According to this technique, the pulse width of the received sensing signal changes as the pH changes. Five different kinds of pH sensitive dyes were used. Methyl red, methyl orange, thymol blue, Nile red and rhodamine-B containing optical-fiber sensing elements were used to fabricate the sensor array. To observe the sensing ability of the proposed sensor array, we measured the buffer solution’s pH from 2 to 12, and we obtained satisfactory and excellent results. The sensitivity of the proposed sensor array was about 0.46 µs/pH, with linear sensing properties over a wide pH range and a correlation coefficient (R^2^) value of approximately 0.997. The proposed optical-fiber sensor array also offered several features including low fabrication costs, high reproducibility performance with a relative standard deviation (RSDs) of about 0.019, highly stable sensing response, reusability, and the capability of remote sensor monitoring. Moreover, the cost of the electronic components used to design the electronic circuitry is low, and the components are available at local electronic suppliers. In future studies, we will use other pH indicators to fabricate optical-fiber sensing elements to increase the number of sensing elements in the array. We also plan to design an optical-fiber probe-type pH sensor, an interdigitated capacitor-based pH sensor array, and an optical-fiber PWM taste sensor array. 

## Figures and Tables

**Table 1:** Three levels of analogy within the electrical and optical-fiber PWM sensing systems.

No. of Stage	Port	PWM System	
		Electrical	Optical-Fiber
1	In	Electrical pulse	Light pulse
2	Control	Voltage/current	Refractive index
3	Out	Electrical pulse	Light pulse

**Table 2:** Composition of the pH-sensing solution to prepare different optical-fiber sensing elements of an array.

Sensor ID	Dye	Polymer	Solvent
S0	-	PVC	DMAC
S1	Methyl red (0.05 g)	PVC	DMAC
S2	Methyl orange (0.05 g)	PVC	DMAC
S3	Thymol blue (0.05 g)	PVC	DMAC
S4	Nile red (0.01 g)	PVC	DMAC
S5	Rhodamine B (0.01 g)	PVC	DMAC

**Table 3:** Statistical data of the proposed optical-fiber PWM pH-sensing elements with methyl red, including sensing membrane in a buffer solution with pH of about 7 for the five observations.

Observation Number	Relative Pulse Width (µs)	Standard Deviation of the Relative Pulse Width
1	1.4	0.019
2	1.423	
3	1.389	
4	1.433	
5	1.399	

**Table 4:** pH measurement data using the commercially available pH-Ion meter S220 and the proposed optical-fiber PWM pH-sensing system under different pH of the unknown buffer solutions.

Observation Number	pH-Ion Meter S220	Proposed Optical-Fiber PWM pH-Sensing System
1	7	7 mV
2	4.6	4.53 mV
3	10	10.09 mV
